# Anxiety and Its Associated Factors During the Initial Phase of the COVID-19 Pandemic in Indonesia

**DOI:** 10.3389/fpsyt.2021.634585

**Published:** 2021-03-10

**Authors:** Gina Anindyajati, Tjhin Wiguna, Belinda Julivia Murtani, Hans Christian, Ngurah Agung Wigantara, Anggi Aviandri Putra, Enjeline Hanafi, Kusuma Minayati, Raden Irawati Ismail, Fransiska Kaligis, Ary I. Savitri, Cuno S. P. M. Uiterwaal, Hervita Diatri

**Affiliations:** ^1^Department of Psychiatry, Faculty of Medicine, Universitas Indonesia—Cipto Mangunkusumo Hospital, Jakarta, Indonesia; ^2^The Center for Clinical Epidemiology and Evidence-Based Medicine Faculty of Medicine, Universitas Indonesia—Cipto Mangunkusumo Hospital, Jakarta, Indonesia; ^3^Julius Global Health/Julius Center for Health Sciences and Primary Care, University Medical Center Utrecht, Utrecht, Netherlands

**Keywords:** anxiety, COVID-19 pandemic, Indonesian, mental health surveillance, psychosocial support

## Abstract

**Introduction:** Coronavirus disease 2019 (COVID-19) is caused by a novel coronavirus which has not been identified previously in humans. The disease leads to respiratory problems, systemic disorders, and death. To stop the virus transmission, physical distancing was strongly implemented, including working and school from home (WFH & SFH). The limitation altered daily routines and needs advanced to adapt. Many have felt uncomfortable and this could have triggered anxiety symptoms. This study aimed to evaluate the proportion of significant anxiety symptoms and its association with COVID-19-related situations in an Indonesian context during the initial months of the pandemic.

**Methods:** An online community survey was distributed through social media and communication platforms, mainly WhatsApp, targeting people >18 years old in Indonesia. Anxiety symptoms were assessed using Generalized Anxiety Disorder-7 (Indonesian Version). Demographical data and information on social situation related to the COVID-19 pandemic were collected. The proportion of clinically significant anxiety symptoms was calculated and the association with demographic and social factors was assessed using chi square test (χ^2^) and logistic regression for multivariate analysis.

**Results:** Out of 1215 subjects that completed the survey, 20.2% (*n* = 245) exhibited significant anxiety symptoms. Several factors, such as age (AOR = 0.933 CI 95% = 0.907–0.96), sex (AOR = 1.612 CI 95% = 1.097–2.369), medical workers (AOR = 0.209 CI 95% = 0.061–0.721), suspected case of COVID-19 (AOR = 1.786 CI 95% = 1.001–3.186), satisfaction level of family support (AOR = 3.052 CI 95% = 1.883–4.946), and satisfaction level of co-workers (AOR = 2.523 CI 95% = 1.395–4.562), were associated with anxiety.

**Conclusion:** One out of five Indonesian people could have suffered from anxiety during the COVID-19 pandemic. The riskiest group being young females, people who had suspected cases of COVID-19, and those with less satisfying social support. Nevertheless, health workers were found to have a lesser risk of developing anxiety. Accessible information and healthcare, social connection, supportive environment, and mental health surveillance are important to prevent bigger psychiatric problems post-pandemic.

## Introduction

Coronaviruses are a group of viruses with a single-stranded RNA structure. Some of the subtypes are known to affect humans, most of which cause mild infections in the upper respiratory tract of immunocompetent individuals (1–3). Some notable once-novel variants of these viruses have caused several outbreaks on a larger scale, such as the severe acute respiratory syndrome (SARS) outbreak in 2002 and the Middle East respiratory syndrome (MERS) in 2012 (2, 3). The common signs and symptoms of coronavirus disease 2019 (COVID-19) infection include acute respiratory symptoms, such as fever, cough, and dyspnea. The disease can lead to pneumonia, acute respiratory distress, acute kidney injury, and even death (4). At the end of January 2020, WHO declared COVID-19 as a public health emergency (5). The virus infection has spread to over 182 countries including Indonesia. The global devastation has well-overtaken the SARS epidemic in 2003, which affected 26 countries and caused 800 deaths worldwide (6). The rising global case numbers are the unprecedented health event of this century. The general public is under increasing strain due to the constant updates from health authorities. Moreover, almost unlimited news coverage from mass media has already been shown to be related to and amplify the level of anxiety across the viewership (7, 8).

In order to minimize the coronavirus infection rates, governments and health authorities of countries worldwide, including Indonesia, have implemented large-scale social restriction measures for various communal activities, including changes in the systems of labor and school from home largely starting as of April 2020 (9). Short-term social restriction might not be a significant problem for the majority of people (10); however, as social beings, extended social restriction may be expected to cause mental health problems. Reflecting on the Indonesian context, where people commonly have a close relationship with extended family and peers, social restriction and the abrupt change to remote working and home-based learning, especially for extended periods, will potentially affect mental well-being.

The mental problems that might be found during a pandemic can be summarized into: (1) stress and anxiety for self and family health, e.g., being transmitted to or transmitting the disease to family or society (11, 12); (2) sleep and eating disorders; (3) attention disturbance; and (4) increased substance use. Stress is a feeling of emotional and physical tension which arises from any event that affects homeostasis (13). While fear of the unknown may cause anxiety, it is a natural bodily response to stress. Acute stress, depression, and anxiety have been rising significantly during the COVID-19 lockdown, and will likely increase when prolonged (12). Therefore, it is crucial to study how alarming the anxiety is in the population, especially in relation to daily life functions (13).

Based on a study in Basque, Spain, anxiety was experienced by all ages, although younger people showed greater stress compared to older people (12). Anxiety is believed to be exacerbated by any exposure of the public to false information that leads to misinterpretations and developing negative thoughts and insecurity (8, 14). These conditions also likely threaten many other groups of people including medical workers (15). Increasing risks to the mental health of medical staff have been reported previously during infectious disease pandemics; PTSD increased by 20% during the SARS epidemic in 2003 (16, 17).

Indonesia Basic Health Research 2018 data showed that about 9.8% of the population has mental and emotional health problems (18). The current COVID-19 pandemic may further raise the proportion of the population that experiences emotional problems. The aim of our study was to evaluate the relationship between anxiety symptoms and the COVID-19 pandemic situation by estimating the current population prevalence of such symptoms. We hypothesized that anxiety level during the COVID-19 pandemic was higher compared to prevalence before the pandemic. In addition, we believed that several factors, for example, socio-demographic characteristics, access to health information, social restriction practice, status of COVID-19 infection, and social support, were related to anxiety. The result of this study could help establish baseline data on mental health in the general population nationwide, and be used as a reference to develop public policy for psychosocial support during a pandemic.

## Methods

### Design

The current study was part of a larger community survey evaluating anxiety during the COVID-19 pandemic in Indonesia. This cross-sectional study was conducted involving the adult Indonesian population from April to May 2020, which was the first and second period of large-scale social restriction implementation. Data were collected through an online questionnaire using the SurveyMonkey program. The survey was distributed through various social media and communication platforms, including WhatsApp groups and personal accounts on Instagram, Twitter, and Facebook as the most commonly used social media by the public (19). Each respondent was asked to give their informed consent before filling out the questionnaire, of which a total of 1,215 adults participated and completed. The protocol of this study was reviewed and approved by the Medical Research Ethics Committee of the Faculty of Medicine Universitas Indonesia (KET-375/UN2.F1/ETIK/PPM.00.02/2020).

### Measurements

Data on respondents' demographic information, social situation related to COVID-19, and anxiety level were collected. Demographic information included age, gender, educational level, occupation, place of current residence, and socio-economic status. Education was classified into lower (elementary/junior/high school) and higher (bachelor/master/doctoral) levels. Socio-economic status was classified into low to lower-middle income and upper-middle to high income groups according to the gross national income classification by the World Bank. All the respondents were also asked about their implementation of working from home and social restriction policy (physical distancing, minimizing outdoor activity, avoiding group gatherings) during the pandemic, current health status of the respondents and their families regarding COVID-19, and their access to related information, such as physical and mental health guidance. The information sources were differentiated into online media, offline media, and direct information from family or friends.

Health status regarding COVID-19 was classified according to the World Health Organization case definition as follows: healthy individual, suspect case, probable case, and confirmed case. A suspect case of COVID-19 applies to individuals who (1) experience acute respiratory illness (fever and at least one respiratory symptoms); (2) have traveled to, or have a residency history in a local transmission area or contact history with confirmed or probable cases during the last 14 days before symptoms onset. A probable case is a suspect case who has obtained inconclusive laboratory test results. A confirmed case is defined as a person who has a confirmed laboratory test result of a COVID-19 infection.

Several questions were aimed to investigate the respondents' behavior changes during the pandemic, along with satisfaction level toward support from family, friends, and colleagues using a single question for each item. Behavior changes were measured on a yes or no scale. Satisfaction toward support system was classified into satisfied and not satisfied. Family members in an Indonesian context are considered not restricted to the nuclear family (a pair of adults and their socially recognized children, regarded as a basic social unit) but also include extended family members, such as grandparents (20). The respondents were asked to describe all of these variables according to their experiences in the last 4 weeks before completing this questionnaire.

The level of anxiety was assessed using the Indonesian version of Generalized Anxiety Disorder-7 (GAD-7). It is a self-report anxiety screening tool for adults (aged 18–95 years old) which was originally developed by Spitzer et al. (21). This instrument was translated into the Indonesian language by Budikayanti et al. (22) and exhibited good validity and reliability (Cronbach's α = 0.867) (22). The scale consists of seven items evaluating how often the respondents were bothered by anxiety symptoms in the last 2 weeks. It is scored on a 4-point Likert scale (0 = not at all, 1 = several days, 2 = more than half the days, 3 = nearly every day). A total score of 8 or greater is considered as corresponding to clinically significant anxiety (23).

### Statistical Analyses

Prevalence of anxiety disorders was estimated based on the proportion of respondents with clinically significant anxiety. Furthermore, we evaluated if socio-demographic characteristics, access to health information, social restriction practice, health status (COVID-19 infection), and social support had associations with the occurrence of anxiety. Univariate logistic regression was first performed to evaluate the unadjusted association between each factor and anxiety disorder. The statistically significant factors (p < 0.05) from the univariable analyses were then evaluated using a multivariate logistic regression. Data analysis was performed using SPSS IBM version 25.

## Results

### Characteristics of Participants

Of the 1,215 respondents included in this study, 245 (20.2%, CI 95% 17.9–22.5) exhibited clinically significant anxiety. The general characteristics of the respondents are shown in Table 1. The median age of respondents was 29 (min–max = 18–88) years old. Two thirds of the respondents were female (*n* = 884, 72.8%) and 1,026 (84.4%) had finished a higher educational level. Respondents mainly came from the western part of Indonesia, including those who lived in DKI Jakarta, West Java, and Banten, and belonged to the upper-middle to high income socio-economic level.

**Table 1:** General characteristics of respondents.

		***n* = 1,215**	**Percentage (%)**
Age (Median; Min–Max)		29 (18–88)	
Sex	Female	884	72.76
	Male	331	27.24
Educational Level	Elementary/Junior/Senior High School	189	15.56
	Bachelor/Master/Doctoral	1,026	84.44
Occupation	Private Employee	366	30.12
	Medical workers	284	23.37
	Students	194	15.97
	Housewife	104	8.56
	Public Employee	97	7.98
	Teacher	77	6.34
	Entrepreneur	76	6.26
	Unemployed	17	1.40
Level of income	Low to lower middle income ( ≤ $3,995/year)	443	36.46
	Upper middle to high income (>$3,995/year)	772	63.54
Marital Status	Single	649	53.42
	Married	534	43.95
	Widow/widower	32	2.63
Domicile	DKI Jakarta	432	35.56
	West Java	249	20.49
	Banten	131	10.78
	East Java	85	7.00
	Central Java	73	6.01
	Riau	34	2.80
	South Sulawesi	26	2.14
	Yogyakarta	25	2.06
	Bali	23	1.89
	Others	138	11.36

Sixty percent of the respondents (*n* = 739) were working from home at the time of the survey and most of them were implementing the social restriction policy. A majority of respondents had access to physical health information regarding COVID-19 (*n* = 1,126, 92.7%), yet only half of them (*n* = 640, 52.7%) received related mental health information. Most respondents reported to be healthy (*n* = 1,132, 93.1%) and only a few (*n* = 38, 3.1%) had family members with suspected cases of COVID-19. The survey showed that respondents felt satisfied with the support they received from family, friends, and colleagues (Table 2).

**Table 2:** Social situation related to COVID-19 in Indonesia.

		***n* = 1,215**	**Percentage (%)**
Access to physical health information about COVID-19	Yes	1,126	92.67
	No	89	7.33
Access to mental health information about COVID-19	Yes	640	52.67
	No	575	47.33
Working from home	Yes	739	60.82
	No	476	39.18
Practicing social restriction	Yes	1,181	97.20
	No	34	2.80
Being a suspect case of COVID-19	Yes	83	6.83
	No	1,132	93.17
Had family member with suspect case of COVID-19	Yes	113	9.30
	No	1,102	90.70
Applying behavior changes due to COVID-19 pandemic	Yes	1,140	93.83
	No	75	6.17
Satisfaction level of family support	Not Satisfied	106	8.72
	Satisfied	1,109	91.28
Satisfaction level of friends support	Not Satisfied	83	6.83
	Satisfied	1,132	93.17
Satisfaction level of co-workers support	Not Satisfied	111	9.14
	Satisfied	1,104	90.86
Clinically significant anxiety (GAD score >8)	Yes	245	20.16
	No	970	79.84

### Factors Associated With Anxiety During the COVID-19 Pandemic in Indonesia

In the univariate analysis, age was statistically significantly related to anxiety. The clinically significant anxiety group had a lower median of age compared to the not-clinically significant anxiety group. Furthermore, several factors, such as sex, level of education, occupation, level of income, and marital status, were found to be related to anxiety. Satisfaction level of support from family, friends, and co-workers was also found to have an association with anxiety (Table 3).

**Table 3:** Analysis of factors associated to anxiety.

		**Clinically significant anxiety**		**Univariable**				**Multivariable**			
		**Yes (*n* = 245)**	**No (*n* = 970)**	**OR**	***p*-Value**	**Confidence Interval 95%**		**AOR**	***p*-Value**	**Confidence Interval 95%**	
						**Lower**	**Upper**			**Lower**	**Upper**
Age (Median; Min–Max)		26 (18–88)	31 (18–81)	–	<0.001^a^	–	–	0,933	<0.001	0.907	0.96
Sex	Female	200	684	1.86	<0.001^b^	1.31	2.64	1.612	0.015	1.097	2.369
	Male (ref)	45	286								
Educational level	Elementary/junior/senior high school	63	126	2.32	<0.001^b^	1.65	3.27	1.235	0.38	0.771	1.977
	Bachelor/master/doctoral (ref)	182	844								
Occupation	Health workers	33	237		<0.001^b^			0.209	0.013	0.061	0.721
	Non-health workers	135	599					0.392	0.138	0.114	1.349
	Students	71	123					0.452	0.191	0.138	1.485
	Unemployed (ref)	6	11								
Level of income	Low to lower middle income	106	337	1.43	0.013^b^	1.08	1.91	0.975	0.877	0.707	1.344
	Upper middle to high income (ref)	139	633								
Marital status	Unmarried	181	500	2.66	<0.001^b^	1.95	3.63	1.128	0.568	0.747	1.702
	Married (ref)	64	470								
Working from home	Yes	144	595	0.90	0.462^b^	0.68	1.12	–			
	No (ref)	101	375								
Practicing social restriction	Yes	236	945	0.69	0.353^b^	0.32	1.51	–			
	No (ref)	9	25								
Suspect case of COVID-19	Yes	22	61	1.47	0.136^b^	0.88	2.45	1.786	0.05	1.001	3.186
	No (ref)	223	909								
Family member with suspect case of COVID-19	Yes	21	92	0.90	0.660^b^	0.55	1.47	–			
	No (ref)	224	878								
Access to physical health information about COVID-19	No	20	69	1.16	0.573^b^	0.69	1.95	–			
	Yes (ref)	224	901								
Access to mental health information about COVID-19	No	110	465	0.89	0.394^b^	0.67	1.17	–			
	Yes (ref)	135	505								
Behavior change due to COVID-19 pandemic	Yes	236	904	1.91	0.069^b^	0.94	3.90	1.993	0.085	0.91	4.365
	No (ref)	9	66								
Satisfaction level of family support	Not satisfied	57	49	5.70	<0.001^b^	3.77	8.61	3.052	0	1.883	4.946
	Satisfied (ref)	188	921								
Satisfaction level of friends support	Not satisfied	43	40	4.95	<0.001^b^	3.14	7.81	1.373	0.362	0.694	2.718
	Satisfied (ref)	202	930								
Satisfaction level of co-workers support	Not satisfied	56	55	4.93	<0.001^b^	3.29	7.38	2.523	0.002	1.395	4.562
	Satisfied (ref)	189	915								

*Constant: −3.381; Hosmer dan Lemeshow test: χ^2^ = 14.631. df = 8, p = 0.067; Nagelkerke R^2^ = 0.190; ref, reference group*.

a *Mann–Whitney test*.

b *χ^2^ (chi-square)*.

Multivariate analysis (Table 3) showed that age was related to clinically significant anxiety, in which older age was found to have a lower risk of developing clinically significant anxiety. In addition, health workers had an almost five times lower chance of developing clinically significant anxiety when compared to those who were unemployed. However, female sex was independently related to the higher possibility of anxiety. A similar trend was seen for being a suspected case of COVID-19. Furthermore, respondents who reported dissatisfaction toward family and co-workers' support had a three- and two-times higher chance of developing clinically significant anxiety, respectively.

## Discussion

This study showed that 20% of our respondents had clinically significant anxiety during the COVID-19 pandemic. Anxiety was more common among those who were female, attained a low educational level, were unmarried, and reported dissatisfaction toward family, friends, and co-workers' support. This was the first study involving nationwide respondents to examine anxiety level and its association with psychosocial factors among the adult population in Indonesia during the COVID-19 pandemic. This was also the first to report anxiety proportion in an Indonesian population.

The study was conducted through an online survey that made it easy and convenient for respondents to participate. However, our study also had limitations due to restriction in eligibility criteria, and relying on respondent's willingness to participate in the survey may lead to selection bias. The majority of respondents were female, of younger age, completed higher education, and had an upper-middle to high socio-economic level; thus, they may not represent the whole Indonesian population. Based on the National Census of 2020, 70% of the Indonesian population are of productive age (15-64 years old), with a balanced number of both females and males, and with only 10.2% who had completed higher education (24).

Similar findings were found in other online studies in Indonesia. The psychosocial aspect of the pandemic also had a majority of female respondents (25, 26), even though both sexes numbers are equal based on the latest national census (27). This study also did not validate the data collected based on certain standards, however our findings were similar with previous research (8, 28). The current study also did not investigate previous common mental disease or previous contact with mental health services. This may have resulted in an overestimation of anxiety prevalence in our study. Internet access provides massive exposure to COVID-19 information, from sources such as social media and online news portals, which may have consequently caused anxiety. Furthermore, a previous study showed that anxiety was more common in the younger age (12). Yet, more research regarding association between socio-economic status and anxiety are needed.

Our findings on anxiety were higher when compared to several studies before the COVID-19 pandemic, including the national number from Indonesia Basic Health Research in 2018 which stated that 9.8% of the adult population had mental and emotional problems (18). Our proportion was also higher when compared to the WHO global prevalence of anxiety, which was 3.6% (29). When compared to other pandemics, such as SARS and MERS-CoV, in different countries, our proportion of anxiety prevalence was relatively higher. A meta-analysis performed by Rogers et al. (3) showed that the point prevalence of anxiety in the SARS outbreak was 14.8% (3). In addition, prevalence of anxiety among people in isolation (due to contact with positive cases) during the MERS outbreak in Korea was around 7.6% (30). However, the prevalence found in this study was relatively lower than the findings in countries like China and Turkey, which revealed anxiety prevalence of 35.1 and 45.1%, respectively (31, 32). The wide gap might be explained by the variation in the timing of the study and the national situation of the COVID-19 pandemic.

Several factors have been identified to play a role in the increase of anxiety during the outbreak, as stated by the World Health Organization (WHO) (33). First, fear of being susceptible to infection was common during the outbreak (34). Second, misinterpretation of perceived bodily sensations and changes could lead to increased episodes of “health anxiety” or hypochondriasis (35). Third, excessive exposure to news related to COVID-19 and misinformation from untrustworthy sources could increase the level of anxiety (33). The news regarding COVID-19 is disseminated continuously through multiple media outlets such as newspapers, television, radio, and social media. People also tend to seek information related to the ongoing event to keep themselves updated, especially during times of crisis (36, 37). Yet, due to inadequate regulation of the information, people can be exposed to misleading information which results in acute stress and intense fear (36–38). Most of the respondents in the study stated that they had access to physical health information about COVID-19. However, only half of them gained mental health information regarding the disease. This provides insight into how many people perceive the current event as something that only affects their physical health. Furthermore, there was insufficient knowledge to support mental well-being during the pandemic due to limited information sources or reluctance to access the information provided. Therefore, special regulations must be made by the government to control the validity and reliability of information given in the media, along with the dissemination of mental health information on how to cope with the stressful circumstances of the pandemic (36–39).

This study also analyzed several factors that could be associated with anxiety level among the general population. Female gender was significantly associated with anxiety in this study. A study from Megatsari et al. (26) also found that young females experienced higher levels of anxiety compared to the male group. Females are known to have a higher risk of developing anxiety compared to males (40) due to both biological [e.g., sex chromosome (41, 42), reproductive hormone exposure (43)] and social factors (44). In addition, several studies about COVID-19 also reported that female gender was significantly related to anxiety (45–47). Over the last few years, most families have both the partners working. However, wives still share a larger responsibility of household chores and childcare (48). In this pandemic, the working from home policy might create a more intense burden for wives, as they need to fulfill their office obligations like attending meetings and completing tasks, at the same time as they need to accompany their children for activities like studying at home.

Marital status was found to be significantly associated with anxiety during the COVID-19 pandemic in the univariate analysis of this research. Unmarried (including single, widow, widower) status was found to increase the risk of anxiety in our study. These findings were in accordance with previous findings (49–51). As many have stated, marriage is commonly known as a protective situation against physical and mental health problems through several mediating factors, such as increased personal and social support, added social status, and stigma reduction (52–54). However, in multivariate analysis, marital status was not significantly associated with anxiety. Our study did not evaluate the marital quality, which might be an important factor acting as a protective feature of marriage (53, 55).

Our findings also showed that lower educational level was significantly related to anxiety at the bivariate level, however it was found to not be significantly related at the multivariate level. This is different to what was shown in previous studies (56–58), although a study in the Indonesian population also found that respondents with secondary and lower education were around three times more likely to exhibit a higher level of anxiety (26). In our study, educational level was not related to anxiety due to the fact that this variable might be greatly influenced by other variables in our study, for example our study subjects were mostly health workers who showed a lower anxiety level in our study. Further study is thus recommended to evaluate educational level and anxiety symptoms during the COVID-19 pandemic in different populations. Several components, such as material, psychological, and behavioral factors, and the existence of chronic disease have been shown to play an important role in lower education related to anxiety (57). Material factors, such as housing tenure, no use of car, insufficient food budget, no private health insurance, and unemployment, were found to have strong connections with anxiety (57).

Our study showed that occupational status was not related to the occurrence of anxiety. However, multivariate analysis described that a healthcare worker was not more anxious than the unemployed. This might be associated with the amount of exposure to COVID-19-related information. They were confident in knowing and managing the symptoms, as well as in prevention practices. In contrast, the unemployed group may not have sufficient knowledge and skills to manage the risk due to lack of exposure (39, 59).

In our study, level of income, which could be included in the material factor, was not significantly related to anxiety in the multivariate analysis. This could be due to the fact that our survey was conducted during the initial period of the large-scale social restriction implementation; therefore, people still thought that the circumstance was only temporary. The huge economic loss had not been felt yet as the businesses had just been shut down for a short period (60). In spite of that, the higher economy class did not show a high prevalence of anxiety, because they might have been more prepared in case of financial problems if they caught COVID-19.

In the present study, the majority of respondents were working from home and executing social restriction measurements. Anxiety may also have resulted from the impact of COVID-19 containment measures. Implementation of quarantine and social restriction policy leads to decreased social interaction which consequently increases loneliness. A study in China at the peak of the pandemic showed that home quarantine was part of the psychosocial stressors that were significantly associated with the development of anxiety and depression (8). Several studies have indicated that lonely individuals are more likely to have anxiety and depression as they do not have adequate social support (37, 39, 61). People have been experiencing emotional exhaustion during this pandemic period and have been unable to unleash their emotions due to the isolation faced in the lockdown (39). It has also been reported that there is an increase in the number of people who experienced loneliness during this pandemic and it was significantly associated with suicidal ideation (62). Suicidal thoughts were found to be associated with level of confidence in controlling the pandemic, sufficient resources to survive during the pandemic, and adequacy of information received from the internet as well as from family (25).

Our study also showed that dissatisfaction toward support from family, friends, and co-workers was associated with anxiety. This observation is in line with the study conducted by Ni et al. (63) which showed the interrelation between social support and anxiety. Several studies have indicated that the increasing number of mental health cases worldwide may potentially develop into another pandemic (64). Certain groups of people were prone to mental health issues and had a lack of support; these included children, elderly, immuno-compromised individuals, and healthcare providers. Particularly, parents commonly undermined their children's distress which led to anxiety (65). An Asian multi-countries study found that people with less satisfactory support had more suicidal thoughts among Indonesian students (25). Nonetheless, family support, especially among nuclear families, could improve during self-quarantine or social restriction. The enlargement of family support during quarantine happened as members shared their feelings more often and showed extensive care toward one another (6, 66). The family factor is expected to play a protective role toward anxiety, as strong bonds with family and the extended kinship network is a common practice in Indonesia. Family becomes the medium to establish intimate relationships in which the members could share feelings, solution to problems, respect, love, and care (67, 68). We presumed that social restriction policy was potentially weakening the social bond, yet nowadays, we can use digital information technology (IT) development as a part of a mitigation scheme to cope with mental health impact. Some popular communication platforms such as online video meetings and social media groups could help maintain mental health support. Healthcare providers extensively use this information technological advance to provide mental health information and telemedicine consultations (63, 64). However, it must be noted that anxiety can also be experienced by healthcare professionals. It is very important to plan special approaches to mitigate anxiety among healthcare workers, as they eventually play an enormous role in resolving anxiety in the community (36, 69).

The study also showed that younger age was related to clinically significant anxiety. This finding corresponds with several studies about COVID-19 and anxiety that showed that the younger population (especially young adults) tended to have more anxiety compared to their older counterparts (12, 26, 67, 70). This phenomenon could be explained by considering that young adults, in the majority, had to adapt to additional changes in their daily routine, including a new online educational or work environment (12). As they are currently in a transition period to becoming older adults, young adults also tend to worry about their future. Delay in academic learning has made college students feel anxious as they assume it may disturb their plan to pursue further academic study. The effect of the pandemic on income, job, or ability to pay loans have also become problems that young adults are concerned about; thus, it may trigger anxiety, especially in this pandemic which includes unstable economic circumstances (8). Furthermore, young adults also use social media more frequently when compared to the older population, which could induce anxiety due to information overload and misinformation (70). The elderly are assumed to be less anxious, are more resilient, and better at emotional management since they have more life experience compared to the youth (28).

This study also revealed that the health status of respondents was related to anxiety. However, data on the anxiety level of patients suspected to have COVID-19 were scarce. One study showed that prevalence of anxiety in suspected cases was not significantly different from the confirmed cases (71). On the contrary, another study showed that subjects suspected with COVID-19 could experience psychological distress due to uncertainty over disease status. Separation from family and loneliness along with negative stigmatization also became other issues during the quarantine period which could worsen psychological health (72, 73). Moreover, the majority of respondents in this research came from a higher education level. Better knowledge is associated with higher anxiety levels (74). In this case, the subjects might have better knowledge regarding COVID-19 risks that could trigger greater anxiety. In addition, health status of family members was found to be unrelated to anxiety. This finding resonated with several previous studies (32, 75, 76). Anxiety might be influenced by several factors, thus further study is needed to obtain a clearer picture particularly in this pandemic circumstance.

This research provided important information that can help develop public policy for managing the COVID-19 pandemic or create a mitigation plan for a disaster/pandemic in the future. The high proportion of anxiety proves that it has been a crucial issue during the pandemic. Proper and continuous measures must be implemented to ensure optimum functioning of society, particularly within high-risk populations (young age, female, suspected case of COVID-19, and those with low quality of psychosocial support). To prevent mental health issues during and post-pandemic, people will need access to information and healthcare, availability of psychosocial support including social connectedness, and a supportive environment.

## Conclusion

One in five people affected by the COVID-19 pandemic in Indonesia may have suffered from anxiety. Based on our findings, the factors associated with anxiety were younger age, female sex, suspected case of COVID-19, and less satisfying social support. Psychosocial support with the emphasis on accessible information and healthcare services, social connection, and a supportive environment in households as well as workplaces, are thought to be important. Nevertheless, health workers were found to have a lesser risk of developing anxiety. Further study on anxiety among people without access to digital technology and diverse age group populations (children, adolescents, and geriatrics) is suggested to fully understand the matter. Furthermore, surveillance regarding the long-term effects of the COVID-19 pandemic on mental health is also needed.

## Data Availability Statement

The raw data supporting the conclusions of this article will be made available by the authors, without undue reservation.

## Ethics Statement

The studies involving human participants were reviewed and approved by the Medical Research Ethics Committee from Faculty of Medicine Universitas Indonesia (KET-375/UN2.F1/ETIK/PPM.00.02/2020). The patients/participants provided their written informed consent to participate in this study.

## Author Contributions

TW, GA, and HD were responsible for the study concept and design. TW, GA, HD, BM, NW, AP, EH, KM, and HC contributed to data or analysis tools. GA, BM, NW, AP, EH, KM, RI, FK, and TW collected the data. GA, BM, HC, EH, and KM performed the data analysis. GA, BM, HC, EH, and KM wrote the manuscript. AS, CU, TW, and HD provided critical revision of the manuscript for intellectual content. All authors critically reviewed and approved the final version of the manuscript submitted for publication.

## Conflict of Interest

The authors declare that the research was conducted in the absence of any commercial or financial relationships that could be construed as a potential conflict of interest.
